# Advanced age is associated with worsened outcomes and a unique genomic response in severely injured patients with hemorrhagic shock

**DOI:** 10.1186/s13054-015-0788-x

**Published:** 2015-03-04

**Authors:** Erin L Vanzant, Rachael E Hilton, Cecilia M Lopez, Jianyi Zhang, Ricardo F Ungaro, Lori F Gentile, Benjamin E Szpila, Ronald V Maier, Joseph Cuschieri, Azra Bihorac, Christiaan Leeuwenburgh, Frederick A Moore, Henry V Baker, Lyle L Moldawer, Scott C Brakenridge, Philip A Efron, The Inflammation and Host Response to Injury Investigators

**Affiliations:** Department of Surgery, Molecular Genetics and Microbiology, University of Florida, PO Box 100245, Gainesville, FL 32610-0245 USA; Department of Surgery, Anesthesia, University of Florida, PO Box 100254, Gainesville, FL 32610-0254 USA; Department of Surgery, Aging and Geriatrics, University of Florida, PO Box 100107, Gainesville, FL 32610 USA; Department of Surgery, University of Florida College of Medicine, PO Box 10019, Gainesville, FL 32610-0019 USA; Department of Surgery, Harborview Medical Center, University of Washington, PO Box 356410, Seattle, WA 98195-6410 USA

## Abstract

**Introduction:**

We wished to characterize the relationship of advanced age to clinical outcomes and to transcriptomic responses after severe blunt traumatic injury with hemorrhagic shock.

**Methods:**

We performed epidemiological, cytokine, and transcriptomic analyses on a prospective, multi-center cohort of 1,928 severely injured patients.

**Results:**

We found that there was no difference in injury severity between the aged (age ≥55, n = 533) and young (age <55, n = 1395) cohorts. However, aged patients had more comorbidities. Advanced age was associated with more severe organ failure, infectious complications, ventilator days, and intensive care unit length of stay, as well as, an increased likelihood of being discharged to skilled nursing or long-term care facilities. Additionally, advanced age was an independent predictor of a complicated recovery and 28-day mortality. Acutely after trauma, blood neutrophil genome-wide expression analysis revealed an attenuated transcriptomic response as compared to the young; this attenuated response was supported by the patients’ plasma cytokine and chemokine concentrations. Later, these patients demonstrated gene expression changes consistent with simultaneous, persistent pro-inflammatory and immunosuppressive states.

**Conclusions:**

We concluded that advanced age is one of the strongest non-injury related risk factors for poor outcomes after severe trauma with hemorrhagic shock and is associated with an altered and unique peripheral leukocyte genomic response. As the general population’s age increases, it will be important to individualize prediction models and therapeutic targets to this high risk cohort.

## Introduction

Severe traumatic injury is responsible for a major percentage of deaths worldwide [1] and elderly patients are thought to have greater morbidity and mortality than their younger counterparts [2]. Severely injured patients who develop multiple organ failure (MOF) often demonstrate a failure in protective immunity [3], and it is presumed that advanced age exacerbates these impairments in immune function [4]. However, there has been a lack of concomitant epidemiologic and genomic data in elderly injured patients to help elucidate these mechanisms and determine their association with clinical outcomes.

The *Inflammation and the Host Response to Injury Collaborative Program* Trauma Glue Grant (GG) was a prospective, multi-institutional observational study with the primary aims of describing the epidemiology, proteomic, and leukocyte genomic response in severely injured burn and trauma patients [5]. The latter consisted of patients who had suffered blunt trauma and who were in hemorrhagic shock without evidence of severe traumatic brain injury (TBI). Analysis of total circulating leukocyte gene expression of these patients illustrated that a so-called genomic storm at the level of the leukocyte transcriptome occurred after traumatic injury, adding further human translational investigative support to the fact that the systemic inflammatory response syndrome (SIRS) and compensatory anti-inflammatory responses (CARS) occurred simultaneously rather than sequentially [6,7]. Patients who exhibited a complicated clinical trajectory, defined as greater than fourteen days of persistent organ dysfunction or death, had exacerbation and prolongation of their transcriptomic response, and failure to return to baseline expression patterns [6]. In addition, a rapid genomic composite score was developed, using 63 select genes, which determine within 12 to 24 hours of injury those patients who are destined to have a complicated clinical trajectory [8,9].

Interestingly, recently published data by our group utilizing murine models of infection and trauma do not completely support this severely exacerbated gene expression pattern in mice of advanced age, although restoration of genomic homeostasis is certainly delayed [10,11]. Although murine and human responses to inflammation are certainly not equivalent at the level of the transcriptome [12], genomic expression patterns in some individual pathways, such as innate immunity, can be well-replicated in mice [13]. In addition, researchers are performing translation data in humans that supports these specific differences in inflammatory responses to injury or infection in the elderly [14].

To date, genomic analyses in this severely injured patient cohort have been carried out primarily on total leukocyte populations, rather than on isolated peripheral polymorphonuclear neutrophils (PMNs), which are the predominant circulating leukocytes after severe injury [6]. In addition, the cohorts from these analyses contained only patients <55 years old. Therefore, the goal of this study was three-fold: (1) determine whether advanced age is associated with increased morbidity and poor clinical outcomes both with standard measures of outcome (that is, 28-day mortality), as well as more recently proposed measures of long-term disposition; (2) characterize the PMN genomic response after severe blunt traumatic injury with hemorrhagic shock, and; (3) determine if the genomic storm identified in younger cohorts is also seen in PMNs from the aged after trauma. We hypothesized that advanced age would be associated with worsened outcomes, and a unique genomic response in severely injured patients with hemorrhagic shock.

## Methods

Approval was obtained from the University of Florida Institutional Review Board to analyze de-identified human data obtained from the GG Trauma Related Database (TRDB) prior to initiation of this study [15]. The clinical protocol and consent forms were reviewed and approved by the central administration site at Massachusetts General Hospital (Institutional Review Board (IRB) MGH Protocol # 2002P001743). In addition, the clinical protocol was reviewed and approved by each of the seven participating clinical sites. In every case, signed informed consent was obtained from the individual patient or their designated legal representative. If informed consent was obtained from the legal representative, the patient was re-consented after they had achieved a clinical state where they could provide informed consent. Based on individual IRBs, the time period required to obtain informed consent from the patient or legal representative varied from 24 hours to within hospitalization due to the vulnerable nature of the patient and their legal representatives. In all cases, the IRBs accepted the argument that patients and their families are often vulnerable in the early post-trauma period, and require some time period to adjust to the severity of the patient’s injury, and provide informed consent.

### Data source and cohort selection

The TRDB contains audited and de-identified data obtained from severely injured trauma patients enrolled from seven level-1 trauma centers between 2001 and 2011 [16]. Inclusion criteria included adult patients (age ≥16 years old), who had been severely injured (injury severity score (ISS) >15) having undergone blunt trauma without severe TBI, and with evidence of hemorrhagic shock (systolic blood pressure (SBP) <90 mmHg or base deficit ≥6 mEq/L, and requiring blood transfusion). In order to address confounding by variations in treatment strategies between centers, clinical standard operating procedures (SOPs) were established and applied to all enrolled patients. Compliance with application of these SOPs was subjected to audit over the course of the study [15,17].

As of October 2013, the TRDB contained detailed, prospectively collected demographic, clinical, and outcomes data on 1,928 patients with blunt trauma and in hemorrhagic shock, who met the criteria for this analysis. These patients were separated into two main cohorts, either advanced age (≥55 years old) or young (<55 years old) for epidemiologic analysis. The age of 55 years was used as the cutoff for several reasons. First, the initial phase of the GG limited enrollment to those <55 years, which was later extended to include all patients over the age of 16 and was used as the original age cutoff by the GG to define the aged population [18]. Second, studies by Demetriades and colleagues analyzing the validity of the trauma and injury severity score (TRISS) methodology and by Sauaia *et al*. looking at early predictors of post injury MOF further supported this cutoff. Both illustrated that being age 55 years or older after trauma was associated with worse outcomes than predicted, even after controlling for other injury factors [19,20]. Using these definitions for young and aged, there were 1,395 and 533 patients in the young and aged cohorts, respectively.

In addition to the trauma patients enrolled for epidemiologic data collection in the TRDB, a subset of 244 severely injured trauma patients, 16 to 90 years old, and an additional 21 healthy controls were enrolled for blood sampling for enriched PMN genomic analysis. When separated based on age, there were 67 aged and 177 young trauma patients. The patients in the GG were also classified based on clinical outcomes into complicated, intermediate or uncomplicated cohorts. In this regard, complicated outcomes were defined as either ICU hospitalization longer than 14 days with evidence of ongoing organ dysfunction, or death [6,15]. Uncomplicated outcomes were defined as those with organ recovery and ICU hospitalization for <5 days. Those who fell between these two classifications were considered intermediate. Therefore, the patients were divided into four distinct groups based on clinical outcomes (either complicated or uncomplicated) and age as follows: complicated aged (n = 25), uncomplicated aged (n = 8), complicated young (n = 42) and uncomplicated young (n = 55).

Next, we attempted to remove confounding in these cohorts due to gender or injury severity by creating matched pairs based on gender, abbreviate injury scores (AIS), as well as, the number and timing of the samples obtained or the death of the patient. The gender of healthy controls was matched to the cohort as closely as possible. Unfortunately, no healthy controls older than 55 years were available. However, the transcriptomic analysis of uncomplicated aged and young patients determined that there was no detectable genomic difference between the two groups. Regardless, this resulted in the creation of a much smaller subset for transcriptomic analysis, which included 4 matched uncomplicated aged and young trauma pairs, as well as 17 matched complicated aged and young trauma pairs. These were compared to 4 and 17 healthy control patients, respectively.

### Clinical outcomes and multivariable logistic regression analysis

Baseline patient demographics, injury severity, fluid and blood product resuscitation parameters, serial laboratory values and multiple clinical outcomes, including complicated recovery and 28-day mortality, were obtained from the TRDB. Ventilator-associated pneumonia (VAP) was used rather than ventilator associated events (VAE), as VAE had not been defined by the CDC at the time of study initiation and was therefore not tracked in the database. Univariate analyses were performed between young and aged cohorts using Fisher’s exact test and the Wilcoxon two-sample test as appropriate.

To determine the role of age as an independent predictor of poor outcome, multivariate stepwise logistic regression models were created using prior known and suspected confounding risk factors, as well as any significant predictive factors identified by univariate analysis. Patients who died within 48 hours of injury were excluded from the complicated outcomes model in order to remove confounding effects of death from irreversible hemorrhagic shock or non-survivable injuries. All patients were included for 28-day mortality modeling. All significance tests were two-sided, with a 0.05 alpha level. Statistical analyses were performed with SAS (v.9.3, Cary, NC, USA).

### Gene expression profile analysis

Of the 1,928 trauma patients enrolled, 244 underwent isolated PMN blood sampling, including aged (n = 67) and young (n = 177) patients. In addition, there were 17 healthy control samples from patients <55 years old. Patients enrolled in the leukocyte subpopulation sampling portion of the study had blood drawn within 12 hours of injury and then subsequently at 1, 4, 7, 14, 21 and 28 days after injury while hospitalized. Circulating PMNs were isolated by positive selection using microfluidic cassettes [21]. Ingenuity System Analysis (IPA®) was used to perform genomic analysis among the trauma patients and healthy controls after RNA extraction and hybridization onto proprietary HH1/2/3 GeneChips™ [22], manufactured by Affymetrix (Santa Clara, CA, USA) specifically for the GG program [21,23].

Three separate analyses were performed as follows: (1) all aged and young trauma patients compared to healthy controls; (2) matched uncomplicated young and uncomplicated aged trauma-patient pairs compared to matched controls; and (3) matched complicated young and complicated aged trauma-patient pairs compared to matched controls. Matching for the latter involved identifying complicated patients from each age group who were the same sex, had the same AIS, had the same number and time point of sample isolation, and had both either survived or died by 28 days. For each of these sets, we identified significant trauma-responsive genes and the difference in the genome-wide expression patterns overall, as well as differences at days 0.5, 1.0, and 4.0 days after injury (*P* <0.001, *F*-test). Leave one out cross-validation was performed to compute the misclassification rate, and a Monte Carlo simulation was conducted to test if the miscalculation rate was significantly better than predicted by chance.

In addition, the datasets were analyzed for individual gene expression differences (magnitude of fold change, *P* <0.001), as well as for individual pathway (Gene Ontology and Biocarta, distance from reference (DFR), *P* <0.05) (22), and functional pathway differences (IPA, *Z*-score (<-2, >2)). A *Z*-score <-2, or >2, represents a significant change at a 95% CI [24].

A secondary analysis of isolated PMN expression using the 63 trauma-responsive genes that had previously been found to be predictive of complicated outcomes after severe trauma [8] was performed and compared in a similar fashion as to above.

To quantify the overall perturbation in gene expression, a modified DFR metric was calculated based on previously reported methodology using the equation. [9]. The DFR is a single natural logarithmic genomic score that summates all the individual gene expression alterations from baseline, whether up- or downregulated, where *e*_*i*_ is the patient’s expression level, and *M*_*i*_ and *V*_*i*_ are the mean and variance of the control group for the *i*^th^ probe set. Thus, each patient’s overall altered transcriptomic response can be represented by a single natural log-transformed metric, although it can also be calculated without the natural log transformation as well. The DFR values for analyzed patients were calculated without taking the natural log of the sums and the results reported are reflective of this.

One-way analysis of variance (ANOVA), Newman-Keul, Kruskal-Wallis, or Holm-Sidak multiple comparison tests were performed when appropriate, with a 0.05 alpha level unless indicated differently above. Genomic statistical analyses were performed using GraphPad Prism 6.00 (La Jolla, CA, USA).

### Plasma cytokine and chemokine analysis

Patients who were enrolled in the leukocyte subpopulation sampling also had plasma samples that were obtained during the study period. Plasma samples were collected within 12 hours of trauma onset, and at 1, 4, 7, 14, 21 and 28 days after injury, or up to the time of hospital discharge if the patient was accessible. Analysis was performed on the 17 matched young and aged trauma-patient pairs with complicated outcomes, along with controls, who were used for the gene expression analysis mentioned previously.

Plasma samples were tested for the following analytes: IL-6, IL-8, IL-10, IL-1β, Interferon gamma-induced protein (IP)-10, Monocyte chemoattractant protein (MCP)-1 and TNF-α, according to the manufacturer’s protocol, on the Luminex MAGPIX® xMap system (Luminex, Austin, TX, USA) using Milliplex® MAP multiplex kits (Millipore, Billerica, MA, USA). The sample concentrations were then generated with the Milliplex® Analyst 5.1 software using best-fit curves.

Two-way ANOVA and generation of general linear models (GLMs) were used for comparison. Because the number of samples in the two cohorts varied over time, whether due to discharge or death over the study period, GLMs were determined to be the appropriate method for comparison. They were used to examine whether the significant differences seen in plasma cytokine/chemokine concentrations were due to age and/or time after injury and the results reported are from this analysis (*P* <0.05, 95% CI). Statistical analysis was performed using SAS (v.9.3, Cary, NC, USA).

The data obtained in the methods and discussed in this publication have been deposited in a public repository, the National Center for Biotechnology Information’s (NCBI) Gene Expression Omnibus [25] and are accessible [GEO:GSE64711] (http://www.ncbi.nlm.nih.gov/geo/query/acc.cgi?acc=GSE64711).

## Results

### Patient clinical characteristics, outcomes and multivariate logistic regression model analysis

The overall cohort consisted of 1,928 severely injured patients in hemorrhagic shock. After dividing the population into young (age <55 years) and aged (age ≥55 years) cohorts, there was no significant difference between the groups in injury severity or total amount of blood transfused (Table 1). Aged patients had an increased number of comorbid conditions at admission, as well as evidence of more severe shock with lower initial SBP, increased lactate levels and higher acute physiology and chronic health evaluation (APACHE) II scores on admission as compared to their younger counterparts (Table 1).

**Table 1:** **Patient demographics and outcomes of patients with severe blunt trauma injury and hemorrhagic shock**

**Patient demographics and outcomes**			
	**Young (age <55 years) (n = 1,395)**	**Aged (age** **≥** **55 years) (n = 533)**	
	**Number (%) or median (IQR)**		***P*** **-value**
Demographics			
Age, years	34 (24 to 45)	66 (59 to 75)	
Gender, male	954 (68%)	331 (62%)	0.010^a^
NISS	36 (27 to 48)	34 (27 to 48)	0.10
Body mass index, kg/m^2^	27 (24 to 31)	28 (25 to 32)	<0.001^a^
Max Apache II score 0 to 24 hours	28 (24 to 32)	32 (27 to 37)	<0.001^a^
Injury to ED arrival, hours	1.3 (0.7 to 2.3)	1.4 (0.8 to 2.9)	0.001^a^
Lowest ED SBP, mmHg	84 (72 to 98)	78 (66 to 88)	<0.001^a^
Max lactate 12 to 24 hours, mmol/L	2.8 (2.0 to 4.3)	3.0 (2.1 to 4.5)	0.035^a^
Total blood 0 to 12 hours, U	4.6 (2.1 to 9.1)	5.0 (2.6 to 9.0)	0.83
Total crystalloid 0 to 12 hours, L	9.9 (7.0 to 14.0)	9.0 (6.1 to 12.7)	<0.001^a^
Major acute surgical procedures	1,312 (94%)	475 (89%)	<0.001^a^
Pre-existing comorbidities			
Preexisting medications	563 (40%)	412 (77%)	<0.001^a^
Major medical comorbidity, ≥1	813 (62.4%)	419 (87.1%)	<0.001^a^
Hypertension	99 (7%)	214 (40%)	<0.001^a^
Congestive heart failure	0 (0%)	0 (0%)	-
Atrial arrhythmia	7 (0.5%)	31 (5.6%)	<0.001^a^
Ventricular arrhythmia	1 (0.1%)	4 (0.8%)	0.009^a^
Peripheral vascular disease	7 (0.5%)	16 (3.0%)	<0.001^a^
Cerebrovascular disease	9 (0.7%)	40 (7.5%)	<0.001^a^
Dementia	0 (0%)	20 (3.8%)	<0.001^a^
Obstructive pulmonary disease	64 (4.6%)	30 (5.6%)	0.35
Malignancy	23 (1.7%)	47 (8.8%)	<0.001^a^
Smoking	423 (30%)	84 (15.8%)	<0.001^a^
Alcoholism	187 (13%)	54 (10%)	0.054
Psychiatric disease	147 (10%)	45 (8%)	0.17
Solid organ transplant	0 (0%)	6 (1.1%)	<0.001^a^
HIV	8 (0.6%)	1 (0.2%)	0.46
Outcomes			
Max Marshall MOF score	4.7 (3.2 to 6.7)	5.3 (3.8 to 7.2)	<0.001^a^
Max Denver 2 MOF score	2 (0 to 3)	2 (1 to 4)	<0.001^a^
On ventilator, days	6 (2 to 13)	8 (3 to 16)	<0.001^a^
Tracheostomy	308 (22%)	145 (27%)	0.019^a^
ICU readmission	137 (10%)	51 (10%)	0.93
Non-infectious complications	592 (42%)	277 (53%)	<0.001^a^
Surgical site infections	202 (15%)	61 (11%)	0.09
VAP	363 (26%)	159 (30%)	0.023^a^
ICU length of stay, days	9 (4 to 17)	11 (5 to 20)	<0.001^a^
Hospital length of stay, days	19 (10 to 31)	18 (9 to 30)	0.07
Complicated outcome	391 (30%)	226 (47%)	<0.001^a^
Mortality at 28 days	54 (6.7%)	76 (15.8%)	<0.001^a^

*P*-value considered significant at <0.05 designated by superscript a. Complicated outcome is defined as >14 days of persistent organ dysfunction or death. NISS, new injury severity scale; ED, emergency department; SBP, systolic blood pressure; MOF, multiple-organ failure; VAP, ventilator-associated pneumonia.

When comparing clinical outcomes among other risk factors, age >55 years was associated with significantly higher MOF scores, longer ICU length of stay (LOS), increased ventilator days, and higher rates of both non-infectious and infectious complications (Table 1). The incidence of VAP and tracheostomy placement was significantly elevated in patients of advanced age. In addition, among other risk factors, age >55 years was associated with significantly higher rates of complicated outcome and 28-day mortality (Table 1). Among those patients who survived, the aged cohort was more likely to be discharged to skilled nursing facilities or long-term acute care facilities, rather than home or rehabilitation facilities (Table 2).

**Table 2:** **Discharge disposition of young and aged trauma patients**

**Patient discharge disposition**			
	**Young (age <55 years) (n = 1,395)**	**Aged (age ≥ 55 years) (n = 533)**	
	**Number (%)**		***P*** **-value**
Home	480 (34.4%)	66 (12.4%)	<0.001^a^
Inpatient rehabilitation	357 (25.5%)	96 (18.0%)	<0.001^a^
LTAC	52 (3.7%)	38 (7.1%)	0.002^a^
Skilled nursing facility	283 (20.3%)	193 (36.2%)	<0.001^a^
Other	43 (3.1%)	12 (2.3%)	0.36
Death (as inpatient)	180 (12.9%)	128 (24.0%)	<0.001^a^

A *P*-value <0.05 was considered significant designated by superscript a. LTAC, long-term acute care facility.

Multivariate logistic regression analysis revealed that age 55 years or older was a strong independent risk factor for the development of a complicated clinical outcome (defined as either ICU hospitalization >14 days with evidence of ongoing organ dysfunction, or death) after controlling for injury severity, shock severity, blood transfusion and comorbidities (Table 3). Similarly, advanced age was a strong independent risk factor for 28-day mortality (Table 3).

**Table 3:** **Multivariate analysis examining the association between age and complicated outcome and mortality after severe blunt trauma**

**Risk factor**	**Odds ratio (95% CI)**	***P*** **-value**
Complicated outcome^1^		
New injury severity score >34	2.82 (2.26 to 3.51)	<0.001
Total blood >9.5 U, 0 to 12 hours	2.72 (2.06 to 3.59)	<0.001
Age ≥55, years	2.25 (1.76 to 2.89)	<0.001
Lactate >6, mmol/L, 0 to 6 hours	1.82 (1.41 to 2.36)	<0.001
Body mass index >28, kg/m^2^	1.56 (1.26 to 1.94)	<0.001
ED SBP <90, mmHg	1.46 (1.16 to 1.85)	<0.001
Crystalloid >12.5, L, 0 to 12 hours	1.43 (1.12 to 1.83)	0.004
Major medical comorbidity, ≥1	1.34 (1.05 to 1.72)	0.019
Mortality at 28 days^2^		
Total Blood > 9.5 (U) 0 to 12 hours	3.91 (2.93 to 5.22)	<0.001
Lactate >6, mmol/L, 0 to 6 hours	2.22 (1.67 to 2.96)	<0.001
Age ≥55, years	2.12 (1.59 to 2.82)	<0.001
New injury severity score >34	1.74 (1.32 to 2.29)	<0.001
ED SBP <90, mmHg	1.66 (1.20 to 2.30)	0.002

^1^Model fit statistics: area under the curve (AUC), *c* = 0.748; Akaike information criterion (AIC) = 1982; likelihood ratio test, *P* <0.0001). ^2^Model fit statistics: AUC, c = 0.775; AIC = 1433; likelihood ratio test, *P* <0.0001). All risk factors were found to be significant, defined as a *P*-value <0.05. Complicated outcome is defined as >14 days of persistent organ dysfunction or death. U, units; ED, emergency department; SBP, systolic blood pressure.

### Neutrophil microarray genomic analysis

Initial genomic analysis demonstrated that there was no significant age-based difference in the transcriptomic expression patterns in the uncomplicated cohorts at 12 hours and one day after injury (*results not shown*). Therefore, further analysis of these groups was not performed.

**Figure 1:**
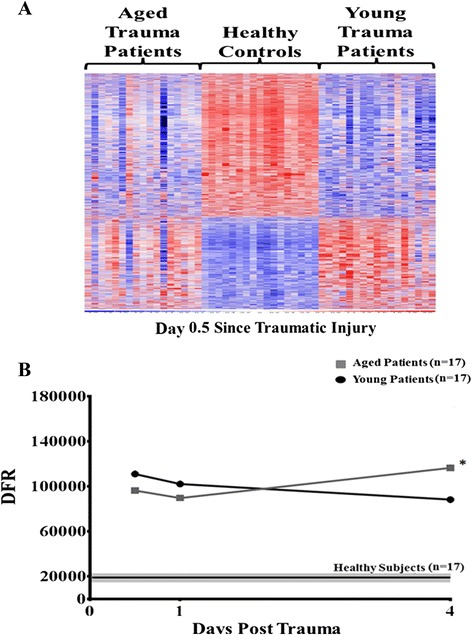
**Heat map and calculated difference from distance from reference (DFR) for polymorphonuclear neutrophil (PMN) genome-wide expression.** Using a false discovery adjusted probability of <0.001 and a two-fold difference in expression, the temporal pattern of the expression of the trauma responsive genes that differed between the matched aged (≥55 years) and young (<55 years old) trauma cohorts with complicated outcomes, as well as healthy controls, is presented. **(A)** Cluster analysis of the cohorts 0.5 days after injury showed that there were 3,121 probe sets (2,095 genes) with expression that was significant expressed among groups (*F*-test, *P* <0.001). In addition, the overall pattern of gene expression was significantly different in each cohort, as determined by leave one out cross-validation. **(B)** Summary of the DFR score calculated for each patient in the complicated aged and young cohorts at days 0.5, one and four days after injury. Analysis revealed significant differences in the DFRs at all the post trauma time points between the two cohorts when compared to controls. In addition, the advanced age cohort had significantly more aberrant gene expression as compared to the young patients on day 4 (Newman-Keuls multiple comparison test, * *P* <0.05).

As our epidemiological analysis illustrated that aged patients had a worse outcome to severe trauma, we wished to determine if there were differences due to age in the transcriptomic response to injury and shock, while trying to exclude the confounders of the magnitude of the injury, gender, the hospital course, and treatment. Thus, we compared the genome-wide expression patterns of matched (for the variables of: complicated outcome; gender; AIS; number of samples isolated at the same time points; and whether the patient died during hospitalization) patients with complicated outcomes. This revealed 3,121 probe sets (2,095 genes) that were differentially expressed between the two age groups at 12 hours after trauma (*F*-test, *P* <0.001). Leave one out cross-validation was used to confirm that the differences in PMN gene expression between young and aged patients could not be explained by chance alone (Figure 1A).

When examining the DFRs calculated for each patient’s PMN gene expression patterns over time, it was observed that both the aged and young cohorts experienced different transcriptomic responses from controls at all measured time points. However, the aged cohort had significantly more aberrant expression 4 days after hospital admission than the younger complicated trauma cohort (DFR (×10^3^): 116 ± 54 versus 88 ± 29 (SD); *P* <0.05) (Figure 1B).

**Figure 2:**
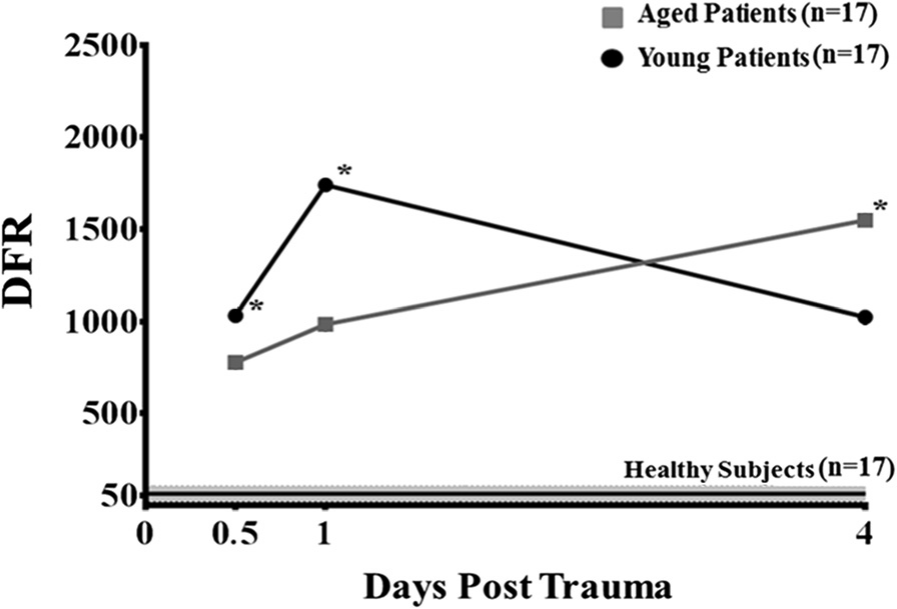
**Calculated difference from reference (DFR) for 51 of the 63 known genes that distinguish clinical trajectory.** Using a false discovery adjusted probability <0.001 and a twofold difference in expression, the temporal pattern of expression of the 51 genes that differed between the matched aged (≥55 years old) and young (<55 years old) trauma patients with complicated outcomes, as well as healthy controls, was analyzed and used to calculate a DFR score. The summary of the DFR scores for the patients in each cohort at days 0.5, 1.0 and 4.0 after traumatic injury is presented. Statistical analysis at 0.5, one and four days revealed significant differences in the DFRs between the young and aged. On days 0.5 and 1.0, the expression patterns in the young complicated trauma patients were significantly more aberrant from control to those seen in the advanced age cohort. By day 4, the expression patterns in the aged were found to be significantly more aberrant from controls than those seen in the young (Newman-Keuls multiple comparison test, * *P* <0.05).

Subsequently, secondary analysis was conducted on the 63 total leukocyte genes whose dysregulation is known to predict complicated outcomes after severe trauma [8]. This demonstrated that only 51 of the 63 previously identified genes were significantly expressed in the PMNs of both the young and aged trauma cohorts with complicated outcomes. Therefore, only these 51 genes were used for subsequent comparison. Analysis demonstrated differential expression over time between the aged and young complicated trauma patients. The DFRs of these 51 genes in the aged cohort with complicated courses, revealed that their gene expression patterns were significantly less perturbed at 12 hours and 1 day after injury (DFR (×10^3^): 1.031 ± 0.364 versus 0.776 ± 390 and 1.741 ± 0.705 versus 0. 984 ± 0.506; *P* <0.05). By day four after injury, the aged patients had significantly increased alterations in their gene expression patterns (DFR (×10^3^): 1.549 ± 0.680 versus 1.024 ± 0.673; *P* <0.05) when compared to controls and their younger counterparts (Figure 2).

Focusing on individual gene fold-changes at 12 hours and 1 day after injury, the young cohort was noted to have significant alterations in gene expression involved in neutrophil chemotaxis (that is, *CCR3, IL-8*), increased inflammation (that is, *HP, MMP8, HMGB1*) and increased responses of the innate and adaptive immune system (that is, *CD24, CD44, IL4R*) when compared to controls. In addition, the young cohort had an increased magnitude of genomic changes, as compared to those of the aged cohort (Table 4). By day 4 post injury, the gene-fold changes seen in the young began to trend back toward control baseline expression, with decreased expression of pro-inflammatory genes and increased immune response (antigen presentation and co-stimulatory molecule genes). The aged cohort, however, continued to demonstrate significant alterations in genes involved in decreased chemotaxis of neutrophils, upregulation of myeloid derived suppressor cells (MDSC), increased inflammation, and decreased response of the innate and adaptive immune system, as compared to controls, 4 days after injury (Table 4).

**Table 4:** **Time-dependent immunity-related gene expression**

**Genes of interest over time**						
**Gene**	**Day 0.5**		**Day 1.0**		**Day 4.0**	
	**Aged**	**Young**	**Aged**	**Young**	**Aged**	**Young**
**Neutrophil chemotaxis**						
*CCR3*	-3.1	-3.6	-3.3	-3.8	-2.7	-1.6
*IL8*	-4.2	-1.8	-12.7	-10.2	-22.5	-7.6
**Immune-related genes/antigen presentation/co-stimulatory molecules/increased MDSCs**						
*ARG1*	6.8	6.9	7.9	7.2	7.4	4.4
*IL4R*	2.9	3.5	3.2	2.7	2.7	2.4
*CD24*	1.9	3.4	-1	2.1	1.8	4.7
*CD44*	2.8	3.9	3.2	3.5		
*OLFM4*	2.6	10.8	1.2	7.1	5.1	7.3
*HERC5*	-3.5	-6.3	-6.1	-9.8	-6	-4.3
*IFIT1*	-4.6	-8.5	-7.9	-14.6	-6.7	-4.3
*IFIT2*	-3.2	-5.7	-4.1	-7.2	-4.4	-2.8
*IFIT3*	-2.3	-3.1	-3.2	-4	-3.7	-2.8
*IFIT5*	-2.6	-3.9	-3.1	-3.6	-2.9	-2.4
*VNN1*	5.4	8	6.3	9.5	6.5	11.2
*IL1R1*	1.8	2	1.4	1.5	1.5	1.4
*IL1R2*	2.1	2	1.7	1.6	1.8	1.5
*HGF*	2.4	3.6	2.7	5.7	2.9	2.7
**Inflammation-related peptides and proteins**						
*CD177*	13.2	21.3	23.3	30.1	18.5	22.3
*HMGB1*	-1.3	-1.5	-1.4	-1.2		
*HP*	5.2	8.9	7.3	11.2	7.8	4.6
*MMP8*	7.6	22.4	5.9	22.6	15.1	9.8
*MMP9*	6.8	9.3	7.8	7.1	7.5	4.5

The table displays the fold changes of selected genes from complicated young and aged trauma patient matched pairs. Positive numbers are indicative of increased fold changes (as compared to controls) and negative numbers are indicative of decreased fold changes. In the acute periods after trauma (days 0.5 and 1.0) the young cohort had greater alterations in gene fold-expression among genes associated with increased inflammation, decreased neutrophil function and impaired immunity as compared to baseline expression in healthy human controls (*P* <0.001) than those seen in the aged. In the sub-acute period (day 4.0), this pattern switched as the young trended toward baseline and the aged continued to experience significantly greater alterations in individual genes from baseline expressions. MDSC, myeloid-derived suppressor cells.

**Figure 3:**
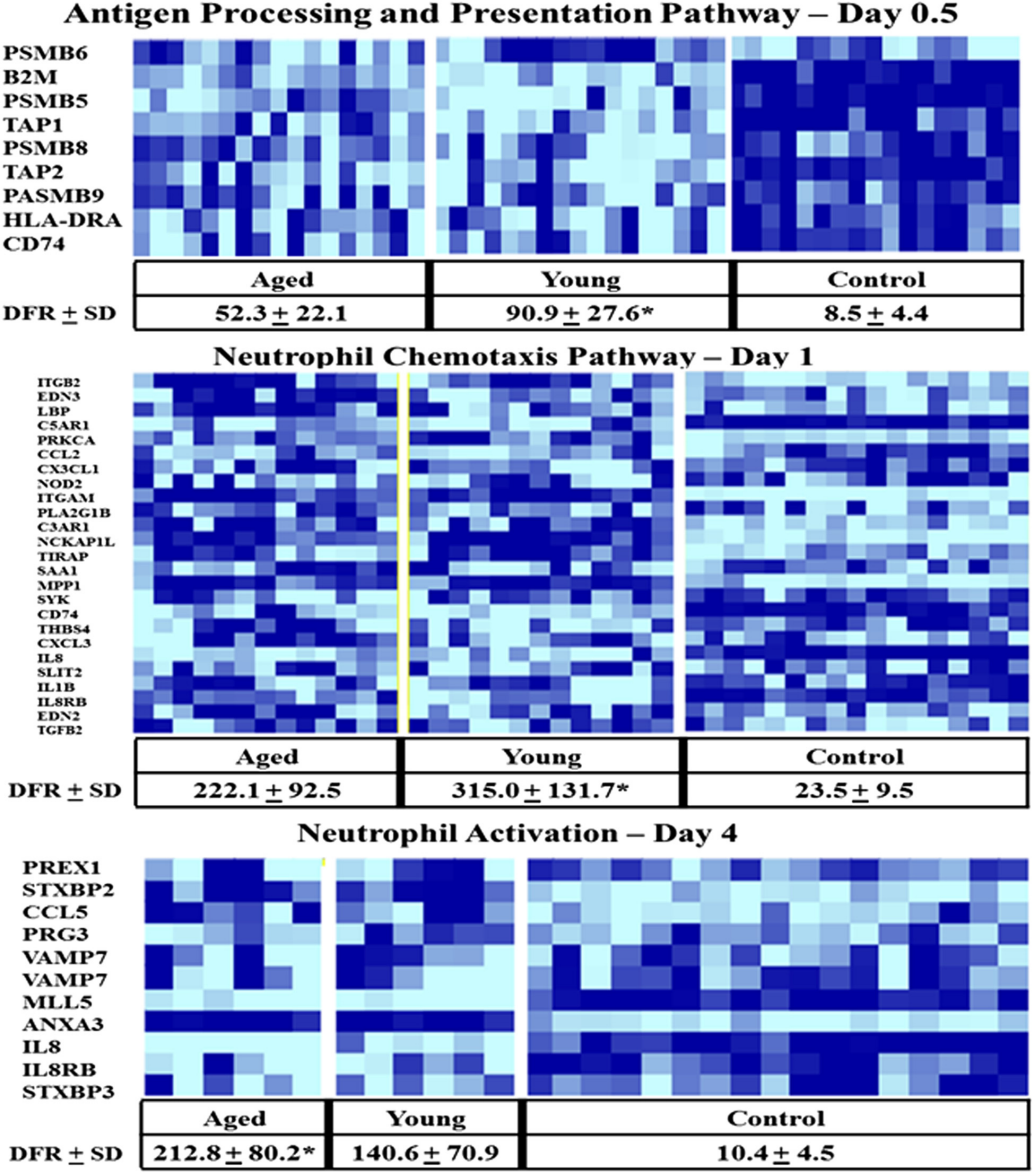
**Selected gene ontology pathway heat maps in complicated aged and young patients on days 0.5, 1.0 and 4.0 after severe traumatic injury.** Dark blue represents upregulation, whereas light blue represents down regulation. In complicated young patients, gene ontology pathway analysis demonstrated that several pathways involved in innate immunity and neutrophil function (that is, antigen processing and presentation and neutrophil chemotaxis pathways) were significantly more aberrant from controls in the acute periods (days 0.5 and 1) than was seen in the aged. In the sub-acute period (day 4) after injury, these patterns switched. The young trended back toward baseline expression values while the aged continued to demonstrate significantly more aberrant expression patterns in pathways involved in innate immunity and neutrophil function (that is, neutrophil activation pathway) (Holm-Sidak, * *P* <0.05).

**Figure 4:**
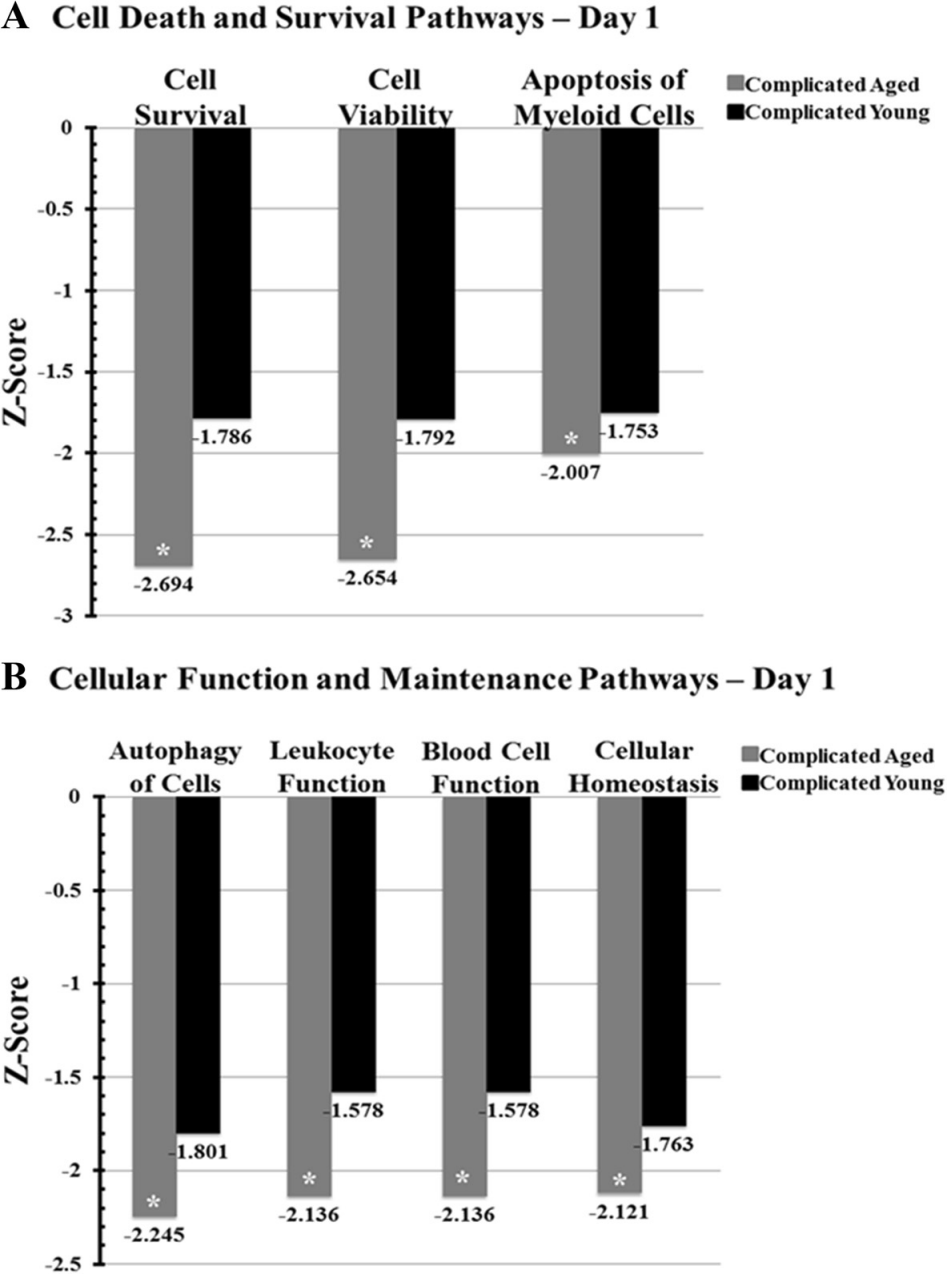
**Selected pathways from functional pathway analysis between complicated aged and young patients after severe traumatic injury.** Functional pathway analysis on day 1 after injury showed that complicated aged patients had significantly downregulated pathways involved in cell survival, function, chemotaxis, immune cell trafficking and hematological system development categories. Graphs display the category broken down into the various subcategories and their corresponding significance level (*Z-score <-2; downregulated). (**A)** Functional analysis on day 1 after severe traumatic injury showed that aged patients had either an upregulation or down regulation of genes leading to a significant overall downregulation of pathways in the cell death and survival category (that is, cell survival, cell viability, apoptosis of myeloid cell pathways) compared to controls. Young complicated traumatic injury patients did not reach a similar significance. (**B)** Similarly, functional analysis on day one showed that aged patients had either an upregulation or downregulation of genes leading to a significant overall downregulation of pathways involved in the cellular function and maintenance category (that is, autophagy of cells, leukocyte and blood cell function and cellular homeostasis pathways) as compared to controls. Again, complicated young trauma patients did not reach a similar significance.

Gene ontology and Biocarta DFR pathway analysis demonstrated that complicated young trauma patients had significantly more aberrant expression patterns (fold change versus control baseline expression) for PMN pathways involved in innate immunity and neutrophil function in the acute period after trauma than those in the aged. By day 4, these patterns once again switched and the aged displayed significantly more aberrant expression patterns compared to controls than the young cohort (Figure 3). Additional IPA® analysis of functional pathways revealed that on day 1 after injury, the aged had significant further downregulation of PMN pathways important to immunity, as compared to the young cohort (Figure 4).

### Plasma cytokine analysis

**Figure 5:**
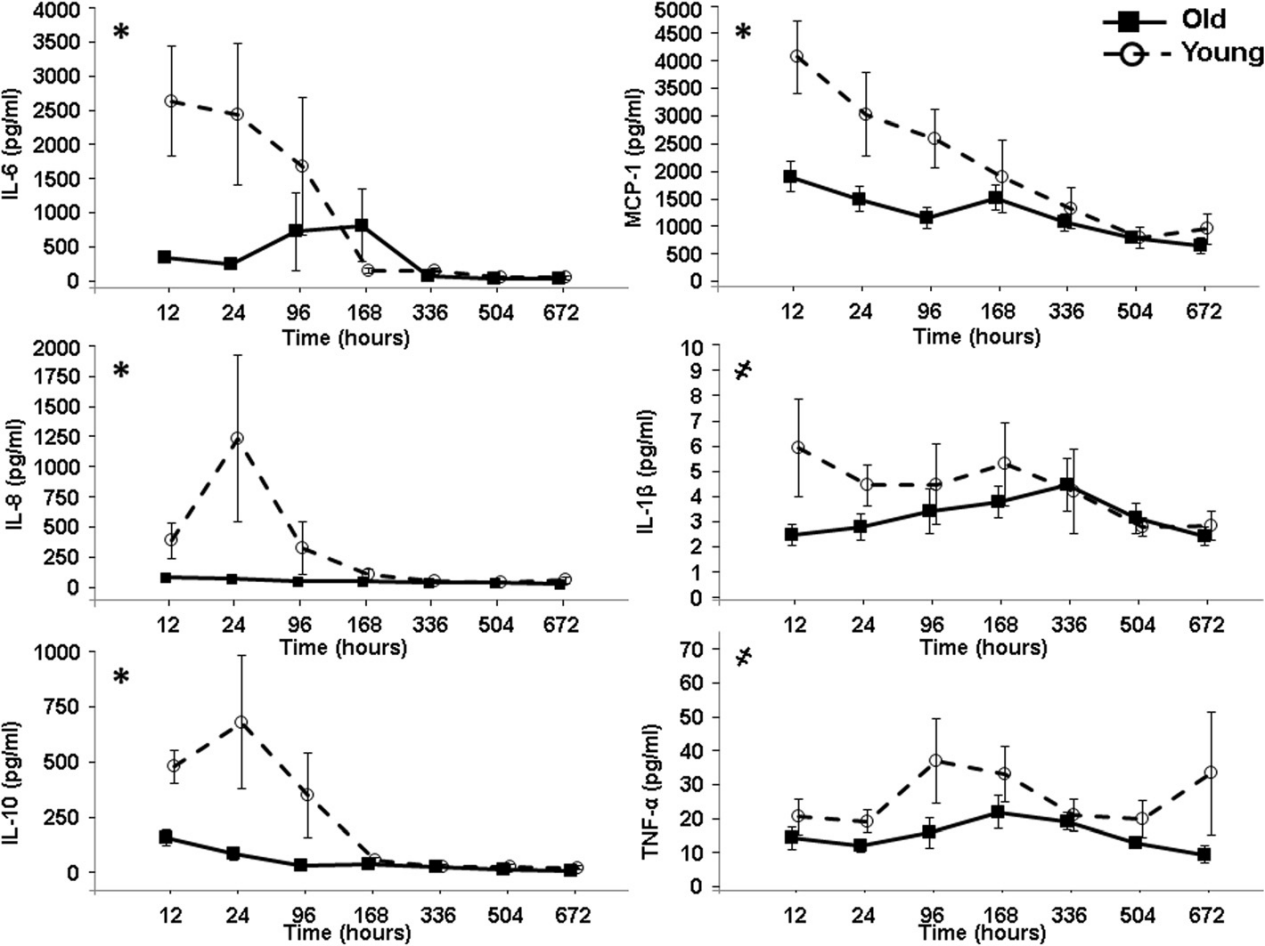
**Plasma cytokine and chemokine levels in complicated aged and young patients at 12, 24, 96, 185, 336, 504 and 672 hours after severe injury and hemorrhage.** The elderly had significantly less cytokine and chemokine concentrations in their plasma after severe blunt trauma. General linear model analysis was performed to examine the significance in relationship of age and time after injury, to the differences seen in the concentrations cytokines and chemokines between the cohorts. Analysis of the plasma demonstrated that both age and time had significant effects on the differences observed for the levels of IL-6, IL-8, IL-10 and MCP-1 (*model *P* <0.05). Model analysis of IL-1β and TNF-α found that only age had a significant effect on the differences observed (^**҂**^ *P* <0.05). Neither age, nor time after traumatic injury, were found to have significant effect on the levels of IP-10 (*data not shown*)*.*

Elderly patients with complicated outcomes had decreased plasma cytokine and chemokine concentrations early after severe hemorrhage and injury, as compared to younger patients with complicated outcomes, verifying our transcriptomic analysis. The elderly had significantly decreased circulating levels of IL-6, IL-8, IL-10, MCP-1, and TNF-α in the acute and subacute periods after severe trauma. No difference was noted for the level of IP-10 (*data not shown*) between the young and aged after severe injury and hemorrhage. GLM analysis showed that these significant differences were found to be a product of both age and time after injury for of IL-6, IL-8, IL-10, MCP-1 and due to age alone for IL-1β and TNF-α (*P* <0.05) (Figure 5).

## Discussion

Advanced age has been shown to be associated with both alterations in immunologic pathways and adverse clinical outcomes after severe trauma [26-35]. The Trauma GG has provided a unique opportunity to examine large amounts of clinical and outcome data. In addition, these data were correlated to genomic data from isolated leukocyte subpopulations in a large subset of patients with advanced age. This analysis confirms, in a prospective, multi-institutional cohort of severely injured patients, that advanced age is an independent risk factor for a complicated course of prolonged organ dysfunction, inpatient mortality, or significant functional disability for those that survive to discharge. Additionally, we have demonstrated that the transcriptomic and cytokine/chemokine response to trauma that results in complicated outcomes is age-dependent, with differential genomic and protein expression of immunologic pathways after injury.

Although advances in surgical critical care have substantially improved early mortality associated with trauma, many patients who survive the initial injury, especially those who are aged, go on to succumb from complications including MOF, secondary nosocomial infections and sepsis [36,37]. Despite decades of promising preclinical and clinical investigations that have elucidated individual aspects of the complex pathophysiology and immunologic disturbances present in sepsis and trauma, our understanding of these entities is still incomplete.

In addition, few therapies targeted towards these mechanistic findings have been successful in improving the outcomes of these chronically critically ill patients [38-40]. Since the elderly population is expanding, research in this cohort has become increasingly relevant, especially with the escalating economic and health care burdens in our society.

In this study, we found that patients over the age of 55 years are more likely to have severe physiologic derangements and increased inpatient mortality after traumatic injury with associated hemorrhagic shock when compared to their younger, similarly injured counterparts. Perhaps most importantly, aged patients who survive the initial insult are more likely to have a prolonged and complicated clinical recovery. These patients then have an increased risk of functional disability at discharge, if one uses their discharge disposition to such places as nursing homes and long-term acute care facilities as a surrogate of their functional status. We believe a complicated clinical course, characterized by an extended ICU LOS and persistent organ dysfunction, are early clinical predictors for patients at risk of developing the persistent inflammation, immunosuppression and catabolism syndrome (PICS) and dismal long-term outpatient outcomes [41].

As previously demonstrated in young human patients, severe traumatic injury is associated with a genomic storm in total circulating leukocytes with concurrent SIRS and CARS responses [6]. In our analysis of the PMN transcriptome and its response to injury in young and aged trauma patients, we found the genomic response patterns in these two populations to be unique. In the acute period after trauma (12 to 24 hours after injury), the PMNs from the young patients display a more aberrant transcriptomic response (increased magnitude) than those with advanced age for both the entire genome and when looking at 51 of the 63 genes previously demonstrated to be predictive of complicated outcomes [8]. This is consistent with immuno-senescence, a state of profound age-associated changes in the immune system, making the aged less capable of mounting an effective immune response [42,43]. This pattern appears to switch in the sub-acute period (day 4 after injury) with continued aberrant genomic expression in the aged cohort’s transcriptome, unlike the young, who appear to trend back towards a homeostatic baseline.

**Figure 6:**
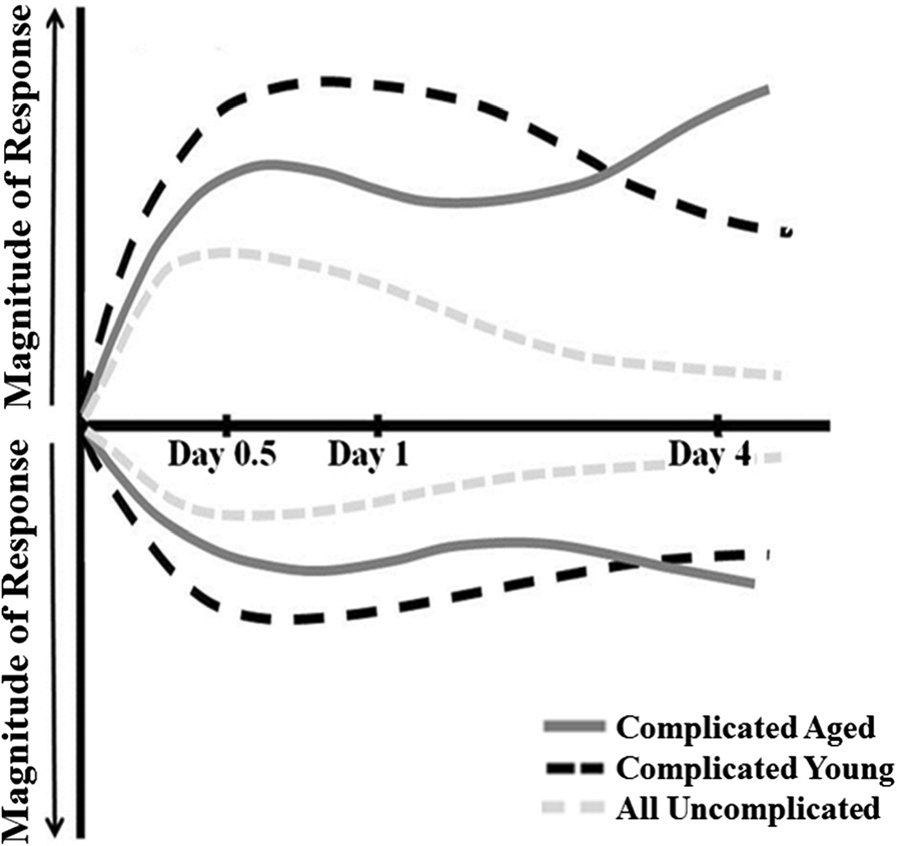
**Our depiction of the summary of the differences in immune response to severe traumatic injury between the young (<55 years old) and the aged (≥55 years old) who experience complicated clinical outcomes.** In the acute period (days 0.5 and 1) after trauma, the aged demonstrate a diminished immune response consistent with immuno-senescence as compared to their younger counterparts. This is followed by continued dysregulation in the advanced age patients as the young trend toward controls by day 4 after injury. In the acute and sub-acute periods after injury, the complicated young and aged trauma patients demonstrate unique genomic expression patterns that are temporal in nature, illustrating that their biologic response to severe injury is different, although they had similar outcomes.

Authors have previously argued that age-related immune dysfunction is due to an exacerbated response in the acute time period to both infectious and non-infectious inflammation [44]. For example, in models of intra-abdominal sepsis, the inflammatory response (as measured by cytokine levels of TNF-α, IL-6, IL-10 and MCP-1) of aged mice was found to be increased as compared to young mice [31]. However, we have published data from murine models to indicate the contrary [10]. Our human analysis of plasma from elderly and young patients with blunt trauma disproves this as well (Figure 5). Additionally, the transcriptomic analysis described in this study indicates that the early PMN response to severe injury and hemorrhagic shock in humans of advanced age is consistent with our murine data, showing that advanced age subjects are unable to mount a robust and effective acute inflammatory response at the level of the transcriptome. This is then followed by persistent aberrant genomic expression patterns that fail to return to baseline in the sub-acute period, suggesting a prolonged state of low-level inflammation (Figure 6).

These findings support our previously described model of PICS [45]. Although inflammaging, an age-related increase in systemic chronic inflammation exists [46], elderly patients, at least at the transcriptomic level, do not have a more exacerbated response to injury as compared to their younger cohorts. In fact, their ability to mount an acute innate immune response is relatively subdued in the acute period. A better comprehension of this phenomenon will be vital as we work towards predictive models of outcome and therapeutic interventions for the injured aged patient population.

Although it is difficult to examine the transcriptomic response related to catabolism in PMNs or other leukocytes isolated from trauma patients, we believe the genes that are significantly altered from baseline expression levels in the aged after trauma do represent the other criteria for PICS: chronic low grade inflammation and immunosuppression [41,45]. As compared to their younger counterparts (Table 4), PMNs from aged patients who had complicated outcomes after severe trauma and hemorrhage revealed continued downregulation of genes required for appropriate leukocyte function (e.g. *CCR3, IL8*) or continued increased expression of inflammatory genes (e.g. *HP, MMP8, MMP*). Although all the genes related to immunity do not follow this pattern, again illustrating the complexity of the leukocyte response to inflammation, we believe that much of the focus of future critical care research will need to be in the PICS phenotype, especially regarding the treatment of the elderly [45].

One of the most interesting genes, from the perspective of PICS and potential interventions, is arginase 1 (Table 4). Although in the acute phase this enzyme plays an important role in myeloid cell antibacterial activity, prolonged expression is associated with the presence of MDSCs [41,45,47]. These cells have suboptimal innate immune responses and are capable of inducing adaptive immune suppression [47]. In the subacute period after trauma, young patients with complicated outcomes being to return to baseline line expression levels of *ARG1* (Table 4) while the elderly continue to have increased transcriptomic expression. It is possible that dysfunctional emergency myelopoiesis in the elderly could lead to increased and prolonged production of MDSCs after inflammation in the elderly, in part explaining their worsening morbidity and mortality after trauma and sepsis. Further study of these cells may reveal them to be a target for immunotherapy to specifically improve outcomes in this population [26-35,41,45,47].

The primary potential weakness of this study is that it is difficult to conclude whether the failure of the PMN transcriptome to return to baseline in the aged is either the result of more frequent complicated outcomes or their cause. One explanation for the continued aberration is the increased frequency of secondary infections and ongoing multiple organ injury. Conversely, the aberrant PMN transcriptome may reflect a circulating neutrophil that is more inflammatory and more immunosuppressed, and it is this aberrant state that contributes to the increased frequency and severity of secondary infections. Although it is likely that both explanations are partially true, interventional studies in animal models and subsequent human clinical trials will be required to fully answer this important question. Second, having an elderly healthy population for comparison would have been beneficial to the study, but was not essential for the evaluation and conclusions. Finally, a higher compliance rate with the clinical SOPs at the various institutions would have been more optimal for this type of study, though over the study period the majority of the SOPs had a compliance rate >69%, and upon review, the application of these SOPs was found to be associated with improved patient mortality during the study period [15]. Although compliance improved over time with repeated audits, there was variable compliance depending upon the bundle audited.

Decades of promising preclinical and clinical investigations have elucidated individual aspects of the complex pathophysiology present after trauma, but our understanding of these entities is still incomplete, and few successful therapies have been introduced to any age group [48,49]. The overall failure of the single mediator or cytokine approach to treat sepsis is actually due, in large part, to an underestimation of the magnitude and diversity of the host response to either severe trauma or infection. Human blunt trauma produces what has been termed a *genomic storm* in which expression of over 75% of the human leukocyte transcriptome is effected [6]. This becomes even more problematic in the aged population, as their leukocyte genomic response is different than that of their younger counterparts, thus indicating that interventional approaches may need to be both multiplex and tailored to different age groups.

## Conclusions

Advanced age is a risk factor for increased morbidity and mortality in many disease processes [26-35]. As the general population’s age increases, this will become more relevant to trauma, which has traditionally been considered more pertinent to younger cohorts. We have found that advanced age is one of the greatest predictors of poor clinical outcomes after severe blunt traumatic injury with hemorrhagic shock, and that advanced aging is associated with a unique genomic expression pattern in circulating neutrophils (Figure 6). The latter challenges many conventionally held beliefs about the elderly response to severe injury, and will have to be taken into consideration when creating individualized prediction models and therapeutic targets tailored to this high-risk cohort.

## Key messages

- After severe trauma, advanced age is associated with more severe organ failure, infectious complications, ventilator days, and ICU LOS, as well an increased likelihood of being discharged to skilled nursing or long-term care facilities.
- Advanced age is an independent predictor of a complicated recovery and 28-day mortality after severe injury and hemorrhage, thus impacting both traditionally analyzed short-term mortality, as well as more recently proposed measures of long-term disposition
- Acutely after trauma, blood neutrophil genome-wide expression analysis of the aged reveals an attenuated transcriptomic response as compared to the young.
- Subacutely after severe injury and hemorrhage, these advanced age patients demonstrate gene expression changes consistent with simultaneous, persistent pro-inflammatory and immunosuppressive states.

## Abbreviations

AIS: abbreviate injury score
ANOVA: analysis of variance
APACHE: acute physiology and chronic health evaluation
CARS: compensatory anti-inflammatory responses
DFR: distance from reference
GG: Glue Grant
GLM: general linear model
IL: interleukin
IRB: Institutional Review Board
ISS: injury severity score
LOS: length of stay
MDSC: myeloid-derived suppressor cells
MOF: multiple organ failure
NISS: new injury severity scale
PMN: polymorphonuclear neutrophil
SBP: systolic blood pressure
SIRS: systemic inflammatory response syndrome
SOP: standard operating procedure
TBI: traumatic brain injury
TNF-α: tumor necrosis factor-α
TRBD: Trauma Related Database
TRISS: trauma and injury severity score
VAE: ventilator-associated event
VAP: ventilator-associated pneumonia
